# Rituximab Combined With Azathioprine for Relapsed Anti‐MDA5 Antibody‐Positive Dermatomyositis With Interstitial Lung Disease: A Case Report

**DOI:** 10.1155/crrh/9772488

**Published:** 2026-10-21

**Authors:** Hiroki Ozaki, Yuki Inoue

**Affiliations:** ^1^ Department of Rheumatology KKR Takamatsu Hospital 4-18 Tenjinmae Takamatsu 760-0018 Kagawa Japan kkr-ta-hp.gr.jp

**Keywords:** anti-MDA5 antibody, azathioprine, dermatomyositis, interstitial lung disease, rituximab

## Abstract

**Background:**

Antimelanoma differentiation‐associated gene 5 (MDA5) antibody‐positive dermatomyositis (MDA5(+) DM) is frequently complicated by interstitial lung disease (ILD) and is associated with a high risk of relapse. Optimal management after relapse remains unclear.

**Case Presentation:**

A 63‐year‐old woman presented with characteristic cutaneous manifestations of dermatomyositis and ILD without muscle involvement. She was diagnosed with MDA5(+) DM complicated by ILD and initially treated with high‐dose glucocorticoids, tacrolimus, and intravenous cyclophosphamide, resulting in clinical improvement. However, after discontinuation of calcineurin inhibitor therapy due to adverse events, disease relapse occurred, characterized by worsening cutaneous lesions, progression of pulmonary infiltrates, and an increase in anti‐MDA5 antibody titers. Subsequently, azathioprine was initiated, and rituximab was administered thereafter. Both clinical and radiological findings improved, and anti‐MDA5 antibody titers progressively decreased. Repeated rituximab infusions were followed by stable clinical findings, allowing glucocorticoid tapering without further relapse.

**Conclusion:**

This case suggests that rituximab combined with azathioprine may be a therapeutic option for disease control after relapse with MDA5(+) DM‐ILD.

## 1. Introduction

Antimelanoma differentiation‐associated gene 5 (MDA5) positive dermatomyositis (MDA5(+) DM) is characterized by distinctive cutaneous manifestations, including ulceration at the site of Gottron’s papules, with or without necrosis, and painful inverse Gottron’s papules on the palmar surfaces. In contrast, it is typically not accompanied by muscle weakness or significant elevations in muscle enzymes. Clinically, MDA5(+) DM is of particular importance because of its strong association with interstitial lung disease (ILD), especially rapidly progressive ILD (RP‐ILD), which represents the leading cause of early mortality. Previous cohort studies have demonstrated that patients with MDA5‐associated RP‐ILD have a substantially poorer prognosis, with markedly lower short‐term survival compared with other myositis associated ILD subsets, such as anti–aminoacyl tRNA synthetase antibody positive disease [1].

Intensive immunosuppressive therapy—consisting of high‐dose glucocorticoid, calcineurin inhibitors (CNI), and intravenous cyclophosphamide (IVCY)—has been shown to improve survival [2]. However, despite aggressive regimens, failure to achieve adequate disease control and relapse remain major challenges. Rituximab (RTX) has been considered a promising rescue or alternative therapy in refractory or relapsed cases. Nevertheless, optimal treatment approaches for patients who experience relapse or intolerance to conventional immunosuppressive agents have not been fully established.

Herein, we report a patient with relapsing MDA5(+) DM‐ILD in whom RTX was used in combination with azathioprine (AZA), resulting in clinical improvement and sustained disease control after relapse.

## 2. Case Presentation

A 63‐year‐old Japanese woman with no significant medical history presented with a three‐week history of skin eruptions and a two‐week history of low‐grade fever and cough. Physical examination revealed fine crackles in both lower lung fields and vasculopathic cutaneous manifestations, including forehead erythema, auricular erythema, painful papular erythema on the lateral and palmar aspects of the fingers (inverse Gottron’s sign), scaly erythema on the elbows (Gottron’s sign), and periungual erythema (Figure 1). No muscle weakness was noted.

**FIGURE 1:**
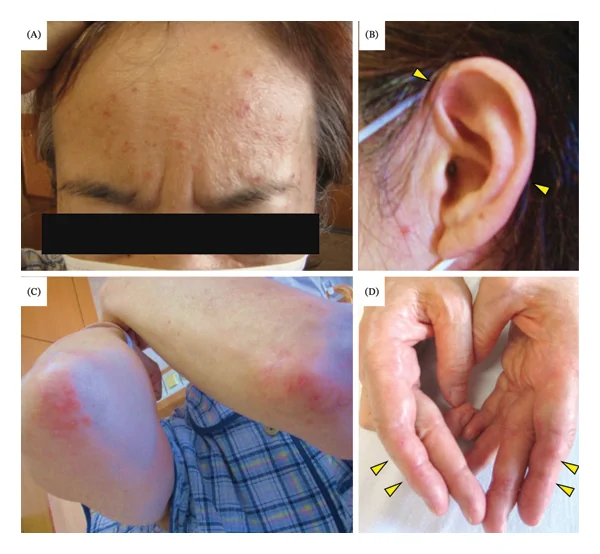
Cutaneous findings of the patient. (A) Red papules on the forehead. (B) Erythema of the left auricle (arrowheads). (C) Gottron’s sign on the extensor surface of the elbow. (D) Periungual erythema and a painful, keratotic papule on the flexor side of the finger known as ‘inverse Gottron’s sign’ (arrowheads).

Laboratory findings showed a leukocyte count of 8840/μL, C‐reactive protein of 1.81 mg/dL, ferritin of 574 ng/mL, KL‐6 of 625 U/mL, and creatine kinase of 41 U/L (Table 1). No muscle weakness was observed, and electromyography and muscle magnetic resonance imaging were not performed. Chest computed tomography (CT) revealed patchy subpleural infiltrates in the right lower lobe (Figure 2A). Forced vital capacity was 89% of predicted. Based on the characteristic cutaneous findings, serological results, and pulmonary involvement, MDA5(+) DM‐ILD was suspected. She was admitted and initiated on induction therapy with prednisolone (PSL; 50 mg/day), tacrolimus (TAC; 3 mg/day), and IVCY (700 mg [500 mg/m^2^]). Anti‐MDA5 antibody levels were later confirmed to be markedly elevated at 3430 index.

**TABLE 1:** Laboratory findings on admission.

Complete blood count		Biochemistry		Immunology	
WBC	8840/μL	CRP	1.81 mg/dL	RF	17 IU/mL
Neutrophils	87.9%	TP	7.4 g/dL	ANA	< 40 titer
Lymphocytes	5.9%	Alb	3.3 g/dL	CH50	52.7 U/mL
Eosinophils	0.7%	AST	43 U/L	Anti‐RNP Ab	< 2.0 U/mL
RBC	390 × 10^4^/μL	ALT	39 U/L	Anti‐SS‐A Ab	< 1.0 U/mL
Ht	34.3%	LDH	219 U/L	Anti‐SS‐B Ab	1.2 U/mL
Hb	11.7 g/dL	CK	41 U/L	PR3‐ANCA	1 IU/mL
Plt	33.2 × 10^4^/μL	ALP	159 U/L	MPO‐ANCA	0.5 IU/mL
		γ‐GTP	43 U/L	Anti‐ARS Ab	< 5.0 index
		BUN	10.7 mg/dL	Anti‐Mi‐2 Ab	< 5 index
		Cr	0.49 mg/dL	Anti‐TIF1‐γ Ab	< 5 index
Arterial blood gas		Ferritin	574 ng/mL	Anti‐MDA5 Ab	3430 index
pH	7.45	HbA1c	6.5%		
pO2	91.5 mmHg	KL‐6	625 U/mL	Urinalysis	
pCO2	37.8 mmHg	β‐D‐glucan	Negative	Occult blood	Negative
HCO3	25.9 mmol/L	T‐SPOT TB	Negative	Protein	Negative

**FIGURE 2:**
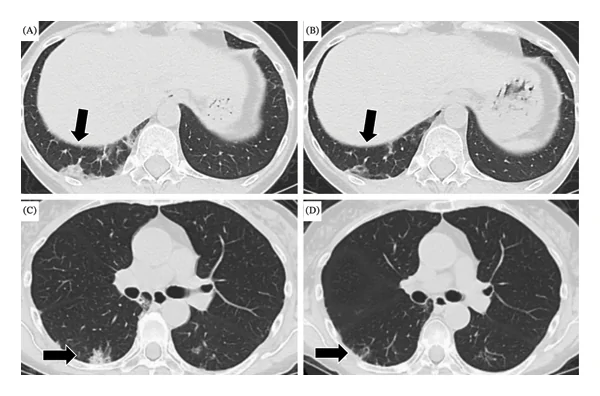
Chest CT findings during the clinical course. (A) Patchy subpleural infiltrates in the right lower lobe at the time of hospitalization (arrow). (B) At 2 months after initial therapy, the consolidation had improved (arrow). (C) At 8 months, recurrence of patchy subpleural consolidation in the right lower lobe (arrow). (D) Following the initiation of RTX and AZA, the consolidation resolved and no recurrence was observed at 34 months (arrow).

The patient’s clinical course is summarized in Figure 3. Following initiation of therapy, her respiratory condition remained stable, and her cutaneous lesions gradually improved. Anti‐MDA5 antibody titers decreased, and follow‐up chest CT showed improvement in pulmonary infiltrates (Figure 2B). A second course of IVCY (850 mg [600 mg/m^2^]) was administered on Day 26. However, cytomegalovirus antigenemia was detected on Day 46 during routine monitoring, although the patient had no symptoms suggestive of cytomegalovirus infection and no evidence of cytomegalovirus‐related organ disease. Further IVCY administration was discontinued after the detection of cytomegalovirus antigenemia. Ganciclovir was administered for 5 weeks until cytomegalovirus antigenemia became negative. Thereafter, she continued treatment with PSL and TAC. Approximately 6 months after treatment initiation, persistent headache and dizziness led to discontinuation of TAC and attempted switch to mycophenolate mofetil (MMF); however, MMF was also discontinued due to recurrent dizziness.

**FIGURE 3:**
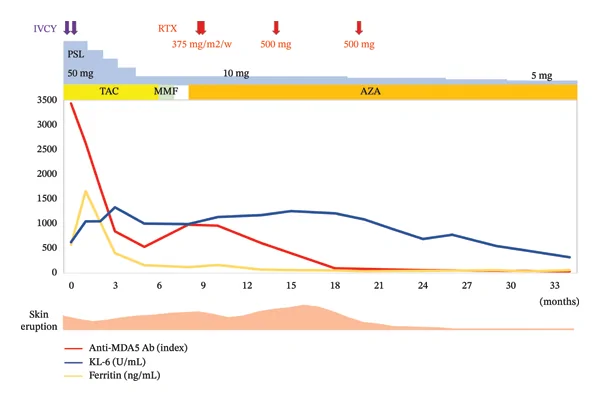
Clinical course of the present case. After initiation of therapy, the patient’s skin eruption improved without respiratory deterioration. Anti‐MDA5 antibody titers decreased, accompanied by radiologic improvement. After two cycles of IVCY, cytomegalovirus antigenemia developed, leading to cessation of IVCY. TAC was discontinued at 6 months due to adverse effects, and MMF was attempted but withdrawn owing to intolerance. During this period, anti‐MDA5 antibody titers remained elevated, with worsening cutaneous and pulmonary findings. Therapy was subsequently switched to AZA combined with RTX, resulting in improvement of pulmonary infiltrates and skin lesions, along with a marked decline in antibody titers. The patient has remained relapse‐free during glucocorticoid tapering. Abbreviations: IVCY: intravenous cyclophosphamide; RTX: rituximab; PSL: prednisolone; TAC: tacrolimus; MMF: mycophenolate mofetil.

Subsequently, disease relapse occurred, characterized by worsening cutaneous lesions, progression of pulmonary infiltrates (Figure 2C), and an increase in anti‐MDA5 antibody titers from 530 to 980 index. Given the relapse with progressive pulmonary involvement and rising anti‐MDA5 antibody titers despite prior immunosuppressive therapy, RTX was selected as a rescue treatment following careful discussion with the patient regarding its off‐label use and potential risks and benefits. Approximately eight months after treatment initiation, AZA (50 mg/day) was initiated and RTX (375 mg/m^2^ weekly for two weeks) was subsequently administered because of disease relapse and intolerance to conventional immunosuppressive agents. One month after RTX, both cutaneous and pulmonary manifestations showed marked improvement. Anti‐MDA5 antibody titers decreased to 605 index by four months. Additional RTX infusions (500 mg each) were administered at six and 12 months after the initial course. By approximately 11 months after RTX initiation, anti‐MDA5 antibody levels had decreased to 58 index, chest CT showed radiologic stabilization (Figure 2D), and glucocorticoids were successfully tapered without further relapse.

## 3. Discussion

MDA5(+) DM is characterized by a high frequency of ILD and a poor prognosis [1]. The clinical manifestations and disease course are heterogeneous, however, and several phenotypic patterns with different risks of RP‐ILD and mortality have been described [2, 3]. Because most disease‐related deaths occur within 6 months of onset, prompt initiation of aggressive immunosuppressive therapy is critical to prevent irreversible pulmonary deterioration. Although early triple‐combination therapy with glucocorticoids, CNI, and IVCY has significantly improved survival, opportunistic infections—particularly cytomegalovirus reactivation—remain common and may lead to treatment interruption or disease relapse [4]. In addition to infectious complications, treatment‐limiting adverse events pose a substantial clinical challenge in the long‐term management of MDA5 antibody‐positive dermatomyositis. Although comprehensive data regarding discontinuation of triple therapy due to adverse events are limited, previous studies of tacrolimus in dermatomyositis and polymyositis, including anti‐MDA5‐positive cases, have reported discontinuation rates of approximately 30% [5]. In the present case, early interruption of immunosuppressive therapy due to cytomegalovirus reactivation and CNI‐related neurotoxicity resulted in subsequent clinical relapse, highlighting the difficulty of maintaining disease control in patients intolerant to standard regimens.

RTX has been reported as a therapeutic option for refractory or relapsed MDA5(+) DM [6, 7]. The RTX in myositis (RIM) trial also evaluated RTX in patients with idiopathic inflammatory myopathies (IIM) [8]. Although the primary endpoint did not differ significantly between the early‐ and late‐treatment groups, more than 80% of patients in both groups demonstrated clinical improvement, supporting the potential efficacy of RTX in refractory IIM. RTX targets the CD20 antigen on B cells and leads to their depletion. By reducing B‐cell numbers, RTX decreases autoantibody production. Experimental studies have suggested that anti‐MDA5 antibodies may contribute directly to ILD pathogenesis [9]. In clinical practice, anti‐MDA5 antibody titers have been shown to correlate with disease activity and prognosis, making them a useful marker for monitoring treatment response [10, 11]. This association is a notable feature of MDA5(+) DM and is not necessarily observed with all myositis‐specific autoantibodies. In the present case, the longitudinal decrease in antibody titers paralleled improvements in both cutaneous and pulmonary manifestations following RTX administration.

Disease relapse remains an important clinical issue in MDA5(+) DM, with reported 5‐year recurrence‐free survival rates of approximately 77% among patients who survive the induction phase [12]. However, optimal management strategies after relapse have not been established. Although several reports have described RTX use in MDA5(+) DM‐ILD, most have focused on rescue therapy for refractory or rapidly progressive disease, and data regarding its role in longer term disease control remain limited [13, 14]. In refractory IIM, a retrospective study reported low‐dose RTX maintenance therapy consisting of a single 1 g infusion every 6 months after induction with standard‐dose RTX, with sustained complete remission in the treated patients [15]. However, the optimal duration and interval of RTX maintenance therapy have not been established. The present case adds to the existing literature by demonstrating that repeated RTX administration can be associated with sustained disease control after relapse, together with parallel improvement in radiologic findings and anti‐MDA5 antibody titers, in a patient who was unable to tolerate standard immunosuppressive regimens.

Long‐term observational data have demonstrated that recurrence rates differ substantially according to the intensity of induction therapy in MDA5(+) DM‐ILD. Patients who achieved remission following intensive induction therapy with a triple combination of glucocorticoids, CNI, and IVCY exhibited excellent long‐term disease control, with a recurrence‐free survival rate of approximately 90% at 10 years, whereas those treated without triple combination therapy showed significantly poorer outcomes, with a corresponding recurrence‐free survival rate of approximately 56% [16]. These findings suggest that insufficient induction therapy may predispose patients to disease recurrence, potentially necessitating continued immunosuppression for long‐term disease control. In the present case, although remission induction with triple combination therapy was initially attempted, treatment could not be continued because of adverse events, resulting in disease relapse. In such clinical scenarios, repeated administration of RTX may be a reasonable therapeutic option for patients with recurrent disease or for those in whom tapering of conventional immunosuppressive agents is difficult. The RTX regimen used in our patient (375 mg/m^2^ weekly for two doses) was lower than commonly used regimens of 375 mg/m^2^ weekly for four doses or 1000 mg twice two weeks apart. However, reduced‐dose RTX regimens have also been reported in MDA5(+) DM, including a regimen of 375 mg/m^2^ on Days 0 and 14, with clinical improvement observed in some patients [17]. In addition, a retrospective study of ANCA‐associated vasculitis reported that 375 mg/m^2^ weekly for two doses achieved clinical outcomes comparable to those of the conventional four‐dose regimen, although this evidence comes from a different disease population [18].

In this patient, RTX was administered in combination with AZA after relapse, which was associated with sustained clinical improvement and stabilization of ILD. In clinical practice, RTX has also been administered with background immunosuppressive therapy in refractory IIM. In the autoimmunity and RTX (AIR) registry, 21 of 30 patients received RTX in combination with at least one immunosuppressive agent, including three patients receiving AZA [19]. However, this observational study did not compare RTX monotherapy with combination therapy and therefore does not establish an additional benefit of concomitant AZA. In our patient, AZA was selected as a tolerable background immunosuppressive agent after tacrolimus and MMF had been discontinued because of intolerance. AZA has a slower onset of action and is not recommended during rapidly progressive phases of MDA5(+) DM‐ILD [20–22]. However, AZA may be appropriate once the disease is stabilized and particularly in patients intolerant to CNI or MMF, as in our case.

This report has several limitations. First, it describes a single patient, and the findings cannot be generalized. Second, the contribution of concomitant immunosuppressive therapy cannot be fully excluded. Larger studies are required to clarify the optimal timing, dosing, and role of RTX in the management of relapsed MDA5(+) DM‐ILD.

In conclusion, this case suggests that RTX may be a therapeutic option for disease control after relapse in the patients with MDA5(+) DM, particularly when standard immunosuppressive therapies are not tolerated.

## Funding

The authors received no financial support for the research, authorship, and/or publication of this article.

## Consent

Written informed consent for publication of this report was obtained from the patient.

## Conflicts of Interest

The authors declare no conflicts of interest.

## Data Availability

Data sharing is not applicable to this article as no datasets were generated or analyzed during the current study.
